# CO_2_ and O_2_ Detection by Electric Field Sensors

**DOI:** 10.3390/s20030668

**Published:** 2020-01-25

**Authors:** Marco Santonico, Alessandro Zompanti, Anna Sabatini, Luca Vollero, Simone Grasso, Carlo Di Mezza, Giorgio Pennazza

**Affiliations:** 1 Unit Of Electronics For Sensor Systems, Department of Science and Technology for humans and the environment, Campus Bio- Medico University of Rome, 00128 Rome, Italy; m.santonico@unicampus.it (M.S.); s.grasso@unicampus.it (S.G.); 2 Unit Of Electronics For Sensor Systems, Department of Engineering, Campus Bio-Medico University of Rome, 00128 Rome, Italy; a.zompanti@unicampus.it (A.Z.); a.sabatini@unicampus.it (A.S.); gingi.dimezza@gmail.com (C.D.M.); 3 Computational Systems and Bioinformatics Lab, Biomedical Engineering Faculty, Campus Bio-Medico University of Rome, 00128 Rome, Italy; l.vollero@unicampus.it

**Keywords:** capacitive sensor, oxygen and carbon dioxide sensors, anthocyanins, electric field, environmental monitoring

## Abstract

In this work an array of chemical sensors for gas detection has been developed, starting with a commercial sensor platform developed by Microchip (GestIC), which is normally used to detect, trace, and classify hand movements in space. The system is based on electric field changes, and in this work, it has been used as mechanism revealing the adsorption of chemical species CO_2_ and O_2_. The system is composed of five electrodes, and their responses were obtained by interfacing the sensors with an acquisition board based on an ATMEGA 328 microprocessor (Atmel MEGA AVR microcontroller). A dedicated measurement chamber was designed and prototyped in acrylonitrile butadiene styrene (ABS) using an Ultimaker3 3D printer. The measurement cell size is 120 × 85 mm. Anthocyanins (red rose) were used as a sensing material in order to functionalize the sensor surface. The sensor was calibrated using different concentrations of oxygen and carbon dioxide, ranging from 5% to 25%, mixed with water vapor in the range from 50% to 90%. The sensor exhibits good repeatability for CO_2_ concentrations. To better understand the sensor response characteristics, sensitivity and resolution were calculated from the response curves at different working points. The sensitivity is in the order of magnitude of tens to hundreds of µV/% for CO_2_, and of µV/% in the case of O_2_. The resolution is in the range of 10^−1^%–10^−3^% for CO_2_, and it is around 10^−1^% for O_2_. The system could be specialized for different fields, for environmental, medical, and food applications.

## 1. Introduction

The detection and the measurement of gas concentrations can be carried out using several principles. The most common working principles, sensors, and systems used for gas detection are: electrochemical sensors [1,2], systems based on Quartz Crystal Microbalances [3], sensors based on semiconductors [4,5], optical sensors [6], capacitive sensors [7], cantilever-based sensors [8], and colorimetric devices [9]. 

Capacitive sensors used as proximity detectors for tracking and control [10] could be a valid option for gas detection, due to their simple structure, which allows miniaturization, their high sensitivity and low cost. In environmental monitoring, indeed, a sensor’s size, simplicity, and cost are strategic to allow a distributed network of sensing elements in order to monitor and map complex scenarios [11,12,13]. 

This networking inter-operability asks for simplicity, which gains reliability for standalone functioning; and low cost and size, which allow wide network building.

Starting from the capacity definition:

$C=\frac{\varepsilon_{0}\varepsilon_{r}A}{d}$ where $\varepsilon_{0}$ is the permittivity in vacuum, $\varepsilon_{r}$ is the relativity permittivity, *A* the electrode area and *d* the distance between the electrodes (dielectric thickness), it could be possible to detect target molecules by inducing a variation of the dielectric layer in terms of modification of *ε_r_* or of its thickness. The latter principle is the most interesting for gas detection.

When the sensor system shows meaningful figures (repeatability, sensitivity, and resolution) which are relevant for the specific application, it can be used in fields such as the monitoring of air quality, or in medicine, for example for the analysis of the exhaled breath. This is the application tested in this work, where a commercial sensor platform developed by Microchip (GestIC), normally used to detect, trace, and classify hand movements in space [14], has been used as an array of chemical sensors. 

It is ambitious to propose a gas sensor which is completely novel (especially for the detection of oxygen and carbon dioxide) with sensitivity, resolution, selectivity, and repeatability better than the ones given by the existing technologies (reported in References [1,2,3,4,5,6,7,8,9,10]). All the mentioned sensors (not pretending to be exhaustive) are complementary in terms of pros and cons, and the best solution can be selected among them for each different application. Thus, the novelty could come from a novel sensing mechanism or from a sensor normally used to detect a different physical quantity, by transforming it into a gas sensor. This is the approach of this study, which finds its motivation in the ambition of exploiting a sensor system that can easily be used in an IoT (Internet of Things) scenario.

## 2. Materials and Methods

**Figure 1:**
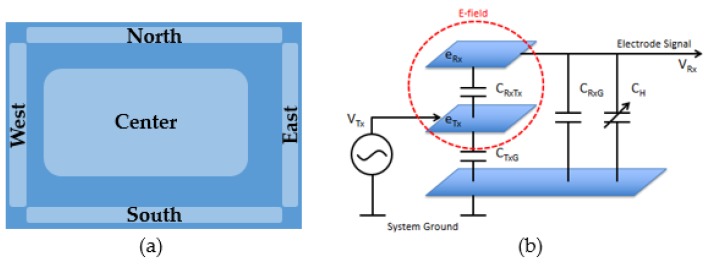
(**a**) Frame Shape electrodes, (**b**) electrode system with characteristic capacitance (V_Tx_: Tx electrode voltage; V_Rx_: Rx electrode voltage; C_RxTx_: capacitance between the receive and transmit electrodes; C_TxG_: capacitance of the transmit (Tx) electrode to the system ground; C_RxG_: capacitance of the receive (Rx) electrode to the system ground; C_H_: capacitance between the receive electrode and the hand (earth ground); e_Rx_: Rx electrode; e_Tx_: Tx electrode).

In this experimental set-up, a capacitive sensor, based on a Microchip Gest-IC platform, has been employed as a gas sensor array in order to measure oxygen and carbon dioxide in different concentrations. The device is based on 3D proximity sensing technology [14], which uses an electric field to detect, trace, and classify hand movements in space. Two overlapped layers comprise the MGC-3030 electrodes system. The first Tx layer shields the ground to allow a propagation of the E-field and grant a better acquisition of the signal. On top of the transmission layer is stacked the Rx layer, which is composed of five electrodes providing spatial resolution. An insulation layer separates the Tx and Rx layers. The five Rx electrodes are placed according to the configuration reported in the Figure 1. 

The responses of the individual electrodes were obtained by interfacing the MGC3030 (I2C serial communication) with an acquisition board based on an ATMEGA 328 microprocessor. A GUI was developed to handle the responses from the five electrodes placed inside the measuring chamber. The interface was developed using MATLAB. 

The measuring chamber was designed using Solidworks and it was fabricated using an Ultimaker3 3D printer. It has dimensions of 120 × 85 mm and it is made of acrylonitrile butadiene styrene (ABS). 

Anthocyanins extracted from red roses [15] were used as a sensing material in order to functionalize the sensor surface. The composition of red rose anthocyanin is widely reported in literature. The most significant amount of red rose anthocyanins was characterized as cyanidin 3,5-di-O-glucoside and pelargonidin 3,5-di-O-glucoside using reversed-phase C18 column chromatography. In particular, the cyanidin 3,5-di-O-glucoside is the predominant constituent, representing about 85% of total content [16].

The results shown in a previous research paper [15], agreed with already-published results.

To obtain a uniform and reproducible bioactive layer, an adhesive mask was placed above the surface of the Rx electrode and it was used to support manual casting by restricting the functionalization only to the exposed area. A volume of 1 mL of the anthocyanin mixture was poured onto the masked surface and it was allowed to air dry at room temperature. Finally, when the solvent was completely evaporated, the adhesive mask was removed.

**Figure 2:**
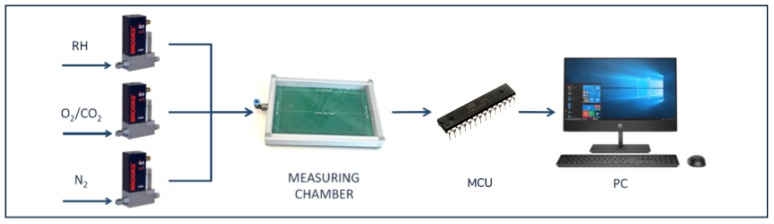
Measurement setup.

The sensor was calibrated using different concentrations of oxygen and carbon dioxide, ranging from 5% to 25%. A flow-meters system was used to control the flow speed and the composition of the gas mixture inside the measuring chamber. The different concentrations were obtained by mixing the gases with inert gas (nitrogen). Nitrogen was also used for sensor recovery after each measurement. The measurement setup is reported in Figure 2.

**Figure 3:**
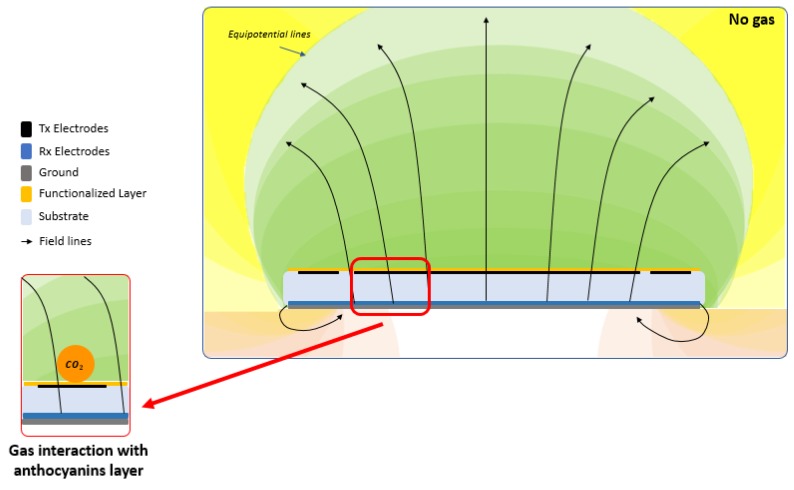
Schematic representation of the sensing mechanism and transduction.

In a second phase, the sensor was calibrated to different O_2_ and CO_2_ concentrations with different humidity levels, from 50% to 90% relative humidity (RH). Each measurement lasted 2 minutes and each recovery phase lasted 5 minutes. Five measurements were executed for each concentration. The output consisted of a voltage signal, which is the result of the electric field perturbation given by the interaction of the sensing material with the gas molecules (see Figure 3). This perturbation is sensed by the electrodes and transduced in a capacitance shift, which is readable by the voltage output of the electronic circuit interface.

## 3. Results

In the initial calibration phase, the sensor was not functionalized. The aim of this initial phase was to explore the sensitivity of the system to the target gases without the presence of a chemically active layer. In these conditions, the system did not show the ability to measure different gas concentrations. 

In the second calibration phase, the central Rx electrode of the sensor was functionalized using anthocyanins (see the Methods section). 

Additionally, in these conditions, the system did not show the ability to measure different gas concentrations. 

Thus, in the third calibration phase, two different mixtures were used:

(1) oxygen, nitrogen, and moisture, ranging from 50% to 90% RH.

(2) carbon dioxide, nitrogen, and moisture, ranging from 50% to 90% RH.

**Figure 4:**
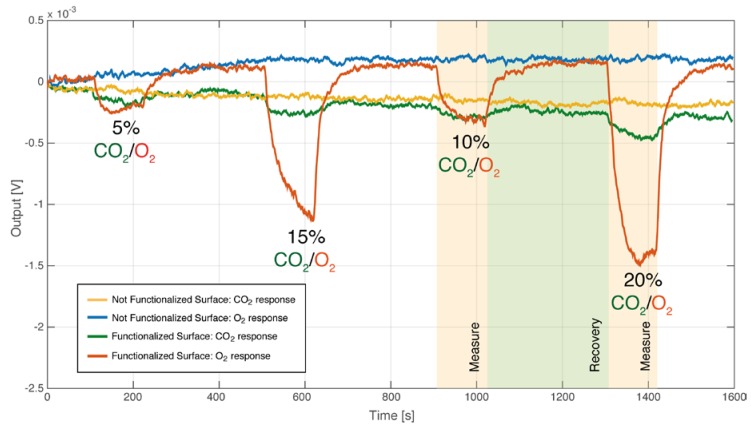
Raw sensor responses are shown: functionalized and non-functionalized sensors were exposed to different CO_2_ and O_2_ concentrations with a fixed relative humidity (RH) level of 50%. Each measurement lasted 2 minutes and each recovery phase lasted 5 minutes.

The raw data relative to the sensor responses are shown in Figure 4.

**Figure 5:**
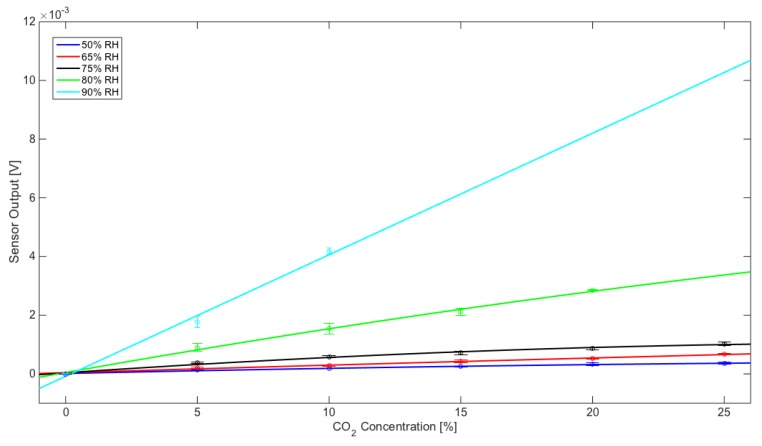
Sensor response to CO_2_: calibration data points were fitted using linear models reported in Table 1. Each error bar has been calculated with mean value and standard deviation based on five measurements.

**Figure 6:**
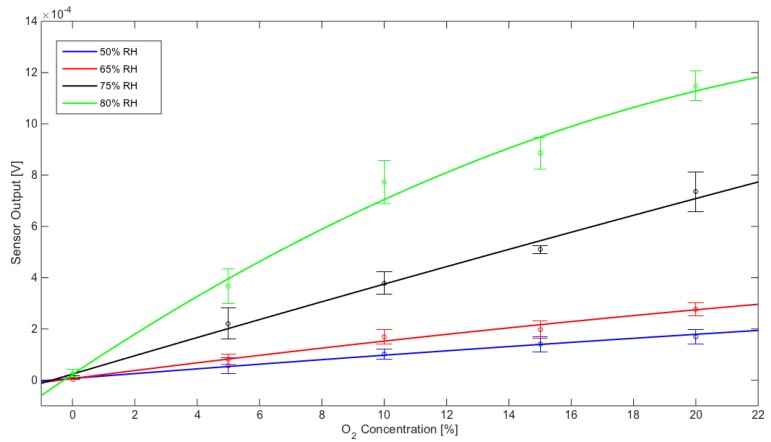
Sensor response to O_2_: calibration data points were fitted using linear models reported in Table 2. Each error bar has been calculated with mean value and standard deviation based on five measurements.

In these conditions, the sensor showed different behaviors for CO_2_ and O_2_ at different moisture concentrations, as displayed by the response curves in Figure 5 and Figure 6. The sensor exhibits a better repeatability with CO_2_ concentrations. 

Curve fitting models for CO_2_ and O_2_ are reported below in Tables S1 and S2 (in the Supplementary Materials). The p1 coefficient always exhibits a far less relevant contribution than p2 and p3, thus all models show a quasi-linear behavior in the concentration range of interest.

To more deeply investigate the sensor response characteristics, sensitivity and resolution were calculated from the curve fitting models at different working points (Table 1 and Table 2).

Sensor performances for CO_2_ and for O_2_ monitoring are summarized below:

- For CO_2_, resolution ranges from about 0.8% at 50% RH to about 0.006% at 90% RH
- For O_2_, resolution ranges from about 0.2% at 50% RH to about 0.05% at 75% RH

According to the performances listed above, the sensor could be exploited in different fields such as: environmental applications, related to the monitoring of air-quality [17]; medical applications, related to the study of the exhaled breath composition and to the monitoring of exhaled breath sampling procedures [18]; and agri-food applications, related to the monitoring of process quality, product packaging, and the assessment of product safety [19]. Moreover, a proper functionalization of the sensor, using an application-specific chemically active layer, will lead to the maximization of the sensing performances, in terms of sensitivity, resolution, and response speed. 

## 4. Discussion

In this paper, a capacitive proximity sensor (which exploits an electric field’s variations) was used as a chemical sensor. The system was calibrated to oxygen and carbon dioxide, functionalizing the central sensing electrode with a biological sensing material. As shown by the response curves, the sensing system was able to measure both carbon dioxide and oxygen concentrations. 

The sensor showed different behaviors to target gases when different moisture concentrations were used. In particular, sensor repeatability was reduced when measuring oxygen concentrations with high moisture levels. To understand this, it is relevant to discuss the role of RH in the sensing mechanism. Potentially, water molecules might act at least at two levels: the first through a direct interaction with gas compounds, and the second by dealing with the anthocyanin sensing layer. In the former case, it is extensively reported by literature that CO_2_ can react with water, forming carbonic acid (H_2_CO_3_), which in turn can release up to two protons (H^+^) per molecule, thus contributing to the generation of the signal. However, the sole RH is not enough to induce a significant electric field modification, as demonstrated by the absence of a signal when the non-functionalized Rx electrode is challenged with both carbon dioxide and oxygen gases. The fundamental role exploited by the anthocyanin sensing layer is indeed to enhance such electric field interference, through one of the many chemical reactivity groups typical of the flavonoid classes. The latter level of interaction is between water molecules and the pyrylium ring (C-ring) of the flavylium ion, which is able to alter the chemical structure of anthocyanins by nucleophilic addition at C2 [20].

On the basis of these results, it will be important to functionalize the system with more sensitive materials to improve sensor performances and allow the measurement of other gases. 

Given the ability of the sensor to measure oxygen and carbon dioxide concentrations in the presence of moisture, an interesting future development could also be the application of this device for the analysis of the human exhaled breath, with the aim of including this device in the measuring chain used for breathprinting [21,22].

This is a pilot study focused on understanding sensors’ relevance in the detection of the selected gases for different applications. The core of the study was the novelty of the sensors (in gas detection), which shows key features for the current sensor technology: low-cost, low-power, and connectivity. No specific applications have been selected here, even if exhaled breath analysis and environmental monitoring have been cited as most attractive. A broad concentration range of CO_2_ has been tested in order to investigate possible applications, also including food production and preservation, incubator atmospheres, CO_2_ entrapment technologies, and monitoring of hazardous sites (mines, etc.). Of course, for a specific application, sensor performances could be improved and tailored in terms of resolution, but the goal of this work was feasibility.

The optimization of the system will allow it to be used in different fields: environmental, medical, and food.

## Figures and Tables

**Table 1:** Resolution and sensitivity of the CO_2_. The V_Noise_ is the fluctuation observed in the output voltage signal in the absence of CO_2_ input for the different experimental conditions of 50%, 65%, 75%, 80%, and 90% of relative humidity.

	50% RH		65% RH		75% RH		80% RH		90% RH	
	Sensitivity	Resolution	Sensitivity	Resolution	Sensitivity	Resolution	Sensitivity	Resolution	Sensitivity	Resolution
%CO_2_	[V/%]	[%]	[V/%]	[%]	[V/%]	[%]	[V/%]	[%]	[V/%]	[%]
5	1.76 × 10^−5^	3.73 × 10^−1^	2.57 × 10^−5^	2.55 × 10^−1^	5.24 × 10^−5^	1.81 × 10^−2^	1.49 × 10^−4^	5.42 × 10^−3^	4.14 × 10^−4^	6.32 × 10^−3^
10	1.52 × 10^−5^	4.32 × 10^−1^	2.50 × 10^−5^	2.62 × 10^−1^	4.31 × 10^−5^	2.20 × 10^−2^	1.38 × 10^−4^	5.84 × 10^−3^	4.14 × 10^−4^	6.32 × 10^−3^
15	1.28 × 10^−5^	5.13 × 10^−1^	2.44 × 10^−5^	2.70 × 10^−1^	3.38 × 10^−5^	2.81 × 10^−2^	1.28 × 10^−4^	6.33 × 10^−3^	-	-
20	1.04 × 10^−5^	6.32 × 10^−1^	2.37 × 10^−5^	2.77 × 10^−1^	2.45 × 10^−5^	3.88 × 10^−2^	1.17 × 10^−4^	6.91 × 10^−3^	-	-
25	7.98 × 10^−6^	8.23 × 10^−1^	2.30 × 10^−5^	2.85 × 10^−1^	1.52 × 10^−5^	6.26 × 10^−2^	-	-	-	-
V_Noise_ [V]	6.56 × 10^−6^		7.91 × 10^−6^		9.50 × 10^−7^		8.08 × 10^−7^		2.61 × 10^−6^	

**Table 2:** Resolution and sensitivity of the O_2_. The V_Noise_ is the fluctuation observed in the output voltage signal in the absence of O_2_ input for the different experimental conditions of 50%, 65%, 75%, 80%, and 90% of relative humidity.

	50% RH		65% RH		75% RH		80% RH	
	Sensitivity	Resolution	Sensitivity	Resolution	Sensitivity	Resolution	Sensitivity	Resolution
%O_2_	[V/%]	[%]	[V/%]	[%]	[V/%]	[%]	[V/%]	[%]
5.0	9.07 × 10^−6^	1.66 × 10^−1^	1.46 × 10^−5^	6.21 × 10^−2^	3.51 × 10^−5^	5.03 × 10^−2^	6.82 × 10^−5^	2.27 × 10^−1^
10.0	8.61 × 10^−6^	1.74 × 10^−1^	1.34 × 10^−5^	6.76 × 10^−2^	3.42 × 10^−5^	5.16 × 10^−2^	5.53 × 10^−5^	2.81 × 10^−1^
15.0	8.15 × 10^−6^	1.84 × 10^−1^	1.22 × 10^−5^	7.41 × 10^−2^	3.34 × 10^−5^	5.29 × 10^−2^	4.24 × 10^−5^	3.66 × 10^−1^
20.0	7.70 × 10^−6^	1.95 × 10^−1^	1.10 × 10^−5^	8.21 × 10^−2^	3.25 × 10^−5^	5.43 × 10^−2^	2.95 × 10^−5^	5.26 × 10^−1^
V_Noise_ [V]	1.50 × 10^−6^		9.05 × 10^−7^		1.76 × 10^−6^		1.55 × 10^−5^	

## Supplementary Materials

The following are available online at https://www.mdpi.com/1424-8220/20/3/668/s1.
