# Supplementary material for: CO2 and O2 Detection by Electric Field Sensors
Source: Sensors (Basel). 2020 Jan 25;20(3):668. doi: 10.3390/s20030668 (PMC7038407; doi:10.3390/s20030668)
Supplement: Supplementary file 1 [file sensors-20-00668-s001.pdf]

Table S1. Curve fitting models for CO_2. The parameters of the fitting curves put in evidence the different contribution of the linear and no-linear terms of the curve. The R-square give an account of the goodness of each fitting option.

RH 50%	RH 65%	RH 75%	RH 80%	RH 90%
p1*x^2+ p2*x+ p3	p1*x^2+ p2*x+ p3	p1*x^2+ p2*x+ p3	p1*x^2+ p2*x+ p3	p5*x + p6
**Coefficients**	**Coefficients**	**Coefficients**	**Coefficients:**	**Coefficients**
p1 = -2.407e-07	p1 = -6.691e-08	p1 = -9.312e-07	p1 = -1.073e-06	p5 = 0.0004141
p2 = 2.001e-05	p2 = 2.637e-05	p2 = 6.175e-05	p2 = 0.0001598	p6 = -8.106e-05
p3 = 1.386e-05	p3 = 4.225e-05	p3 = 3.729e-05	p3 = 4.724e-05	
**Goodness of fit:**	**Goodness of fit:**	**Goodness of fit:**	**Goodness of fit:**	**Goodness of fit:**
R-square: 0.9626	R-square: 0.9795	R-square: 0.9796	R-square: 0.9874	R-square: 0.985

Table S2. Curve fitting models for O_2. The parameters of the fitting curves put in evidence the different contribution of the linear and no-linear terms of the curve. The R-square give an account of the goodness of each fitting option.

RH 50%	RH 65%	RH 75%	RH 80%
p1*x^2 + p2*x + p3	p1*x^2 + p2*x + p3	p1*x^2 + p2*x + p3	p1*x^2 + p2*x + p3
**Coefficients:**	**Coefficients:**	**Coefficients:**	**Coefficients:**
p1 = -4.571e-08	p1 = -1.19e-07	p1 = -8.538e-08	p1 = -1.291e-06
p2 = 9.525e-06	p2 = 1.579e-05	p2 = 3.595e-05	p2 = 8.111e-05
p3 = 6.732e-06	p3 = 6.368e-06	p3 = 2.377e-05	p3 = 2.247e-05
**Goodness of fit:**	**Goodness of fit:**	**Goodness of fit:**	**Goodness of fit:**
R-square: 0.8937	R-square: 0.9486	R-square: 0.9627	R-square: 0.9715
